# A rapid genotyping method for an obligate fungal pathogen, *Puccinia striiformis *f.sp. *tritici*, based on DNA extraction from infected leaf and Multiplex PCR genotyping

**DOI:** 10.1186/1756-0500-4-240

**Published:** 2011-07-20

**Authors:** Sajid Ali, Angélique Gautier, Marc Leconte, Jérôme Enjalbert, Claude de Vallavieille-Pope

**Affiliations:** 1 UMR1290 BIOGER-CPP, INRA-AgroParisTech, BP01, 78850 Thiverval-Grignon, France; 2 UMR 320 Génétique Végétale, INRA, Ferme du Moulon, 91190 Gif sur Yvette, France

## Abstract

**Background:**

*Puccinia striiformis *f.sp. *tritici *(PST), an obligate fungal pathogen causing wheat yellow/stripe rust, a serious disease, has been used to understand the evolution of crop pathogen using molecular markers. However, numerous questions regarding its evolutionary history and recent migration routes still remains to be addressed, which need the genotyping of a large number of isolates, a process that is limited by both DNA extraction and genotyping methods. To address the two issues, we developed here a method for direct DNA extraction from infected leaves combined with optimized SSR multiplexing.

**Findings:**

We report here an efficient protocol for direct fungal DNA extraction from infected leaves, avoiding the costly and time consuming step of spore multiplication. The genotyping strategy we propose, amplified a total of 20 SSRs in three Multiplex PCR reactions, which were highly polymorphic and were able to differentiate different PST populations with high efficiency and accuracy.

**Conclusion:**

These two developments enabled a genotyping strategy that could contribute to the development of molecular epidemiology of yellow rust disease, both at a regional or worldwide scale.

## Background

*Puccinia striiformis *f.sp. *tritici *(PST), an obligate basidiomycete that causes wheat yellow/stripe rust, a serious disease in all major wheat growing regions [1-5]. The development of different molecular markers has aided the description of possible PST migration patterns [6], the emergence of high temperature-adapted strains [7,8] and the existence of recombination [9,10]. Despite these recent developments, numerous questions still need to be addressed, e.g. the evolutionary history of PST, its centre of origin, its historic migration pathways or more recent migrations causing new epidemics. These studies necessitate the genotyping of a large number of isolates, a process that is limited by both DNA extraction and genotyping methods.

Two or three cycles of PST spore multiplication on plants are usually necessary after sampling before DNA extraction. Because of its obligate nature, PST cannot be cultured on routine media to obtain sufficient biomass for DNA extraction [11]. Spore production may be further complicated when dealing with exotic isolates, which involve the mandatory use of expensive, time-consuming and wholly-contained facilities. In addition, using a given set of susceptible varieties to increase the spores of exotic isolates may give rise to bias. We had previously observed very low levels of infection or even resistance reactions in previously considered fully susceptible varieties such as cv. Victo [12], Michigan Amber and Cartago when inoculated with Pakistani isolates. This can result in the loss of isolates having avirulence factors recognized by unknown resistance genes in varieties used to increase spores. One alternative is to extract DNA from one or few spores, and then increase it through a whole genome multiple displacement amplification [11] before performing genotyping. However, the sophistication required for this method, as well as the need to prevent any contamination from other organisms, limits its use. We therefore tested here a third procedure, i.e. the direct extraction of fungal and plant DNA from single spore-infected leaf.

Another issue was the availability of a set of molecular markers sufficient to describe the population structure of a pathogen. The use of microsatellites/simple sequence repeat (SSR) markers with co-dominance and high polymorphism is of considerable value to the study of dikaryotic fungi such as PST [13]. When SSR detection and allele sizing are performed using an automated DNA fragment analyzer based on the separation of fluorescently-labeled amplicons, accurate and efficient genotyping can be achieved [14]. A good way to further enhance the efficiency of SSR genotyping is to multiplex SSR amplifications. Multiplex PCR refers to the simultaneous amplification of several markers in a single reaction, thereby saving the time and money required to manage each PCR reaction separately [15]. This method has been reported as achieving the same specificity as single conventional PCR reactions [14]. We report here a protocol that enabled the amplification of a set of highly informative SSR markers for PST studies, by means of three multiplex PCR reactions for PST.

## Findings

### Improvements to PST DNA extraction

**Figure 1:**
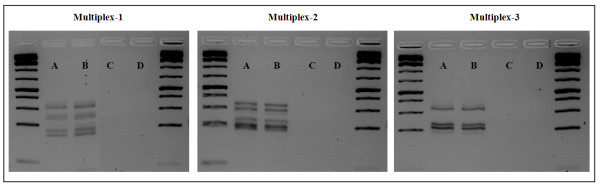
**Gel run for the amplification of three multiplexes for DNA extracted directly from spores and from leaves infected with PST**. A: J1023M2 spore extracted, B: DNA extracted from plant infected with J1023M2, C: Non-infected cv. Michigan Amber seedling leaf DNA, D: Non-infected cv. Sogood adult plant DNA.

To extract DNA from infected leaves, we selected both sporulating leaves from the first cycle of spore multiplication and leaves sampled in the field with single sporulating lesion, i.e. infections presumably resulting from a single spore infection. A Qiagen DNeasy^® ^plant mini DNA extraction kit protocol was used to extract DNA from the sample. The infected leaves were put in 1.5 μL Eppendorf tubes containing 70 μL lysis (AP-1) solution (65°C) and one tungsten bead, and then ground for 3 min at 30 rps with a Retsch-MM300 grinder. An additional 70 μL of lysis (AP-1) solution was added and the liquid material was transferred into special tubes for extraction using the Qiagen DNA 'Biorobot 3000' extraction robot. Further details regarding the protocol are available in the manual (available at http://www.qiagen.com). The DNA thus extracted was then quantified on Nanodrop spectrophotometer (Thermo Scientific) and stored at -20°C until further use for molecular genotyping. The quantity of DNA was within the range 2 ng μL^-1 ^to 6 ng μL^-1^, depending on the size of the leaf lesion. It is essential to remember that these extracts were containing the DNA of both the plant and the fungus. Three PCR multiplex reactions (targeting 20 SSRs, see below) were used to determine whether the PST DNA was of sufficiently good quality for genotyping. The gel pictures for the three multiplexes run with DNA extracted from both the spores and the infected leaves are shown in Figure 1. Although the DNA extractions from infected leaves produced smaller quantities of PCR-amplicons, the amplifications were always sufficient to produce unambiguous signals for all loci when run on a sequencer. To have an idea about the relative amount of PST DNA in wheat DNA, we made a dilution of DNA extracted from PST infected lesion and DNA extracted from pure PST spores. In case of infected leaf extracted DNA, a dilution of two times resulted in no or very weak amplifications, while for DNA extracted from PST spores, a dilution of 20 times was necessary to reach to the lowest concentrations for PCR amplification. This indicates that the infected leaf DNA contains 1/5 of fungal DNA and 4/5 of leaf DNA. Thus at least 3 μL of the infected leaf extracted DNA must be taken to have enough PCR amplification. Further dilution will reduce the amplification to be able to read the alleles unambiguously. Extraction made from infected leaves of different ages, different varieties or with different infection times gave equal amplification. However, the quantity of the infection lesion is important. An infection lesion of at least 2 cm long must be used to have enough quantity of pathogen spores and hyphae for DNA extraction and subsequent amplification. Indeed, the amplification of DNA extracted from non-infected leaves from two wheat varieties (Michigan Amber and Sogood) did not achieve any amplification (Figure 1). The extractions from infected leaves were first validated in four different isolates (two from France and two from Pakistan), for which SSR typing was strictly identical to the genotypes obtained from the DNA extracted from spores (data not shown). This technique was then applied to a set of more than 100 isolates sampled in Pakistan in 2010 which displayed little or no spore production during multiplication. The SSR amplification was highly efficient, while allelic patterns were matching those of isolates sampled at the same location but genotyped after DNA extraction from spores (data not shown). This technique could also be used for single sporulating lesion infected leaves sampled in the field as such an infection normally results from a single spore and has the same genotype. However, in the case of leaves infected with several sporulating lesions, they need to be cloned to obtain single spore lesions that can then be used in the same manner as discussed above. In both cases however, at least half of the sporulating lesions must be preserved in order to retrieve the isolate if it is required in the future. One option would be to isolate samples through mono-chlorosis isolation or single spore inoculation, and then use the infected leaf for DNA extraction while at the same time retrieving the spores for future pathotyping and other biological/epidemiological studies. This direct use of infected leaves for DNA extraction is applicable for any wheat pathogen while considering the purity of races per lesion, eliminating the costly and critical step of exotic isolate multiplication in full confinement.

### Development of an efficient and multiplexed set of SSR for the PST population study

**Figure 2:**
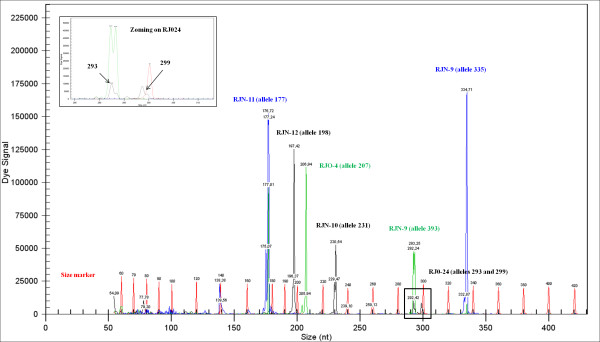
**Chromatogram of Multiplex-2 with seven SSR loci labelled with blue (2 SSRs), green (2SSRs) and black (3 SSRs) fluorescent dyes, while the red dye represents the length markers**. The RJO-24 locus with a low strength peak could be read after zooming (top left).

Another aim of this study was to obtain a reliable combination of different SSR markers that could be amplified during a small number of PCR reactions, rather than a separate PCR amplification for each SSR. We benefited from a total set of 22 previously developed SSRs [13,16] and a set of 13 SSRs sequences kindly provided by Dr. X. Chen (Washington State University, Pullman, USA), out of which only nine were amplified and two were polymorphic. Amplifications were performed first of all on each individual SSR and they were then combined in three multiplexes as a function of their allele sizes. Based on their polymorphism (assessed using a set of eight isolates representing different genetic groups of global PST populations) and after selection for a successful amplification in multiplex PCR reactions, a total of 20 SSRs were chosen. For each SSR locus, the forward primer was labelled with black, green or blue florescent dyes, the red fluorescent dye being reserved for the size marker (Figure 2). SSR loci in the same or closed allele size range were then labelled with different florescent dyes to achieve the maximum possible number of loci per run of the sequencer. Table 1 provides details on the SSRs combined in each multiplex PCR reaction.

**Table 1:** Description of three PCR multiplexes enabling the genotyping of 20 *Puccinia striiformis *f.sp. *tritici *SSR

Multiplex	SSR Locus	Florescence	Allele size range	Reference
**Multiplex 1**	RJN5	Black	225-231	[13]
	RJN6	Black	310-322	[13]
	RJN13	Green	150-153	[13]
	RJN3	Green	339-347	[13]
	RJO21	Blue	167-182	[16]
	RJN4	Blue	258-264	[13]
	RJN8	Blue	306-318	[13]
	RJO18	Blue	334-360	[16]

**Multiplex 2**	RJN12	Black	192-200	[13]
	RJN10	Black	225-231	[13]
	RJO24	Black	273-308	[16]
	RJO4	Green	201-207	[16]
	RJO20	Green	282-293	[16]
	RJN11	Blue	173-185	[13]
	RJN9	Blue	335-337	[13]

**Multiplex 3**	RJN2	Black	172-196	[13]
	WU6	Black	211-213	Provided by X. Chen
	RJO3	Green	202-204	[16]
	WU12	Green	325-334	Provided by X. Chen
	RJO27	Blue	217-243	[16]

The PCR reactions were performed using a QIAGEN kit containing a single mix of Taq-polymerase, MgCl, dNTPs and buffer, referred to as the Type-it microsatellite kit specially designed for Multiplex PCR reactions. Each reaction contained 2 μL water, 1 μL Q-solution, 1 μL of the primer mix (containing 2 μM of each SSR primer), 5 μL of the Type-it mix and 1 μL (15 ng) of DNA. The amount of DNA was increased to 3 μL and no water was added for infected plant leaves, as the PST DNA was diluted with plant DNA. An optimization step was performed to identify the optimum melting temperature for all the SSRs in a given Multiplex. The optimum PCR conditions thus determined were the same for all three multiplexes, with preheating at 95°C for 5 min followed by 30 cycles of 95°C for 30 s, 57°C for 90 s and 72°C for 30 s, with a final extension step at 60°C for 30 minutes, using an iCycler (Biorad) thermocycler. The PCR products were run on a 2% agarose gel to reveal the amplification products. The amounts of PCR product added to 35 μL of the Sample Loading Solution, containing 0.4 μL Beckman Coulter 400-bp size standard, varied as follows:1.5 μL for Multiplex-1 and Multiplex-3 and 2.2 for Multiplex-2 in the case of spore DNA; 3 μL for Multiplex-1 and Multiplex-2 and 4 μL for Multiplex-2 in the case of infected leaf DNA. Amplicon fragments were separated using a Beckman Coulter CEQ-8000 DNA Analyzer with the default FRAG-3 run method. The fragments were read using CEQ-8000 Genetic Analysis System Software (Beckman Coulter) to record alleles manually for each locus according to amplicon fragment lengths. All the alleles were readable and there was no difference between allele lengths whether the PCR was performed for each SSR separately or in a multiplex reaction. Allele sizes were within the range of previously reported alleles for previously developed SSR markers [13,16], while for two new SSRs, the allele sizes were within the range of 211-213 bp for WU-6 and 325-334 bp for WU-12. The technique was then used to genotype more than a thousand isolates representing the worldwide PST population and we found no ambiguity in terms of allele reading, together with an efficient discriminating power for this set of SSRs. The data on these worldwide set of isolates would be used to infer about the PST phylogeny and evolutionary biology. The development of this multiplex-based amplification technique, together with the reading of allele length through a sequencer, achieved gains in time and accuracy, as well as regarding the reproducibility of the results.

## Conclusions

The extraction of DNA from infected leaves, together with a Multiplex-based PCR reaction and the reading of fluorescently labeled alleles through a sequencer thus provides a ready-to-use method for the efficient genotyping of PST, and enables clear gains in terms of time, money, reproducibility and accuracy. Because of the high level of polymorphism of the SSR markers selected, the proposed SSR set could also constitute a genotyping reference at worldwide level, enabling the rapid comparison of genetic analyses. Such easily comparable sets of markers constitute an essential tool for molecular epidemiology and to trace emerging races in a fungus that is known for its highly efficient long distance migration [17]. Furthermore, the genotyping of large number of isolates from different geographical regions coupled with recent population genetics analyses would assist to address ancestral relationship between different geographically spaced populations, describe the ancient migration routes and the role of host and geography on pathogen population structuring. This will help us to understand overall evolution of pathogens and to consequently orientate disease management strategies.

## Competing interests

The authors declare that they have no competing interests.

## Authors' contributions

SA, AG, CP and JE designed the study; SA, ML and AG carried out the experimental work; SA, JE and CP prepared the manuscript. All authors have read and approved the manuscript.
